# Epidemiology of repeat influenza infection in Queensland, Australia, 2005–2017

**DOI:** 10.1017/S0950268822001157

**Published:** 2022-07-18

**Authors:** Olivia Price, Frances A. Birrell, Edin J. Mifsud, Sheena G. Sullivan

**Affiliations:** 1 WHO Collaborating Centre for Reference and Research on Influenza, Royal Melbourne Hospital, at the Peter Doherty Institute for Infection and Immunity, Melbourne, Victoria, Australia; 2 Communicable Diseases Branch, Department of Health, Queensland Government, Brisbane, Queensland, Australia; 3 Department of Microbiology and Immunology, The University of Melbourne, at the Peter Doherty Institute for Infection and Immunity, Melbourne, Victoria, Australia; 4 Department of Infectious Diseases, The University of Melbourne, at the Peter Doherty Institute for Infection and Immunity, Melbourne, Victoria, Australia

**Keywords:** Epidemiology, influenza, recurrence, reinfection, survival analysis

## Abstract

Natural infection with the influenza virus is believed to generate cross-protective immunity across both types and subtypes. However, less is known about the persistence of this immunity and thus the susceptibility of individuals to repeat infection. We used 13 years (2005–2017) of surveillance data from Queensland, Australia, to describe the incidence and distribution of repeat influenza infections. Consecutive infections that occurred within 14 days of prior infection were considered a mixed infection; those that occurred more than 14 days later were considered separate (repeat) infections. Kaplan-Meier plots were used to investigate the probability of reinfection over time and the Prentice, Williams and Peterson extension of the Cox proportional hazards model was used to assess the association of age and gender with reinfection. Among the 188 392 notifications received during 2005–2017, 6165 were consecutively notified for the same individual (3.3% of notifications), and 2958 were mixed infections (1.6%). Overall, the probability of reinfection was low: the cumulative incidence was <1% after one year, 4.6% after five years, and 9.6% after ten years. The majority of consecutive infections were the result of two type A infections (43%) and were most common among females (adjusted hazard ratio (aHR): 1.15, 95% confidence interval (CI) 1.09–1.21), children aged less than 5 years (relative to adults aged 18–64 years aHR: 1.58, 95% CI 1.47–1.70) and older adults aged at least 65 years (aHR: 1.35; 95% CI 1.24–1.47). Our study suggests consecutive infections are possible but rare. These findings have implications for our understanding of population immunity to influenza.

## Introduction

Influenza viruses cause seasonal epidemics that result in considerable morbidity and mortality [1]. Two influenza types, A and B, are recognised to cause significant disease in humans [2]. Currently, two influenza A subtypes (A(H1N1) and A(H3N2)) and two influenza B lineages (B/Victoria and B/Yamagata) co-circulate at varying magnitudes each year. The immune response generated by natural infection with influenza is believed to be cross-protective across both influenza types and subtypes, largely due to the stimulation of a T cell response that targets conserved regions of the virus genome [3–5]. However, less is known about the persistence of this immunity, and thus the propensity for individuals to be reinfected with influenza.

Retrospective analyses of influenza surveillance data suggest that reinfection within a season [6] and over consecutive seasons [7] is perhaps more common than expected. Homologous reinfection (i.e. reinfection with the same subtype or lineage) of immunocompetent children and adults within a year of prior infection has been recorded in multiple longitudinal cohorts [8–13] and case studies [14, 15]. These observations highlight the limited duration of protection afforded by natural infection as a consequence of antigenic drift [12] and also underscore the high variability in individual immune responses post-influenza infection [16]. Improved accessibility and sensitivity of molecular testing for influenza have also led to increased detection of co-infection by multiple influenza viruses [17–22]. The susceptibility of individuals to reinfection and co-infection has implications for our understanding of population immunity and therefore, influenza epidemiology and modelling.

Influenza is a notifiable disease in Australia, meaning laboratory-confirmed infections must be reported to the public health department in each jurisdiction. In this study, we analysed 13 years (2005–2017) of influenza surveillance data from Queensland, Australia, to investigate the incidence of influenza reinfection and mixed infections, as well as the interval between consecutive infections and whether this differs by age group or gender. Such data may inform our understanding about cross-protection and longevity of immunity afforded by influenza infection.

## Methods

### Data source and study population

Influenza notification data from 2005 to 2017 were obtained from the Queensland Department of Health. Influenza infections were confirmed using molecular assay, antigen detection test, virus isolation or serology. The data were de-identified but included a unique person identifier, gender, date of birth, onset date and notification date. Data were cleaned to remove untyped notifications and consolidate notifications made for the same person with different type/subtype (i.e. mixed infection).

We defined consecutive infection as an influenza notification for the same person more than 14 days after a previous influenza notification, as this is when the shedding of the virus of the initial infection is likely to have ceased [23, 24]. In this study, we denote consecutive infections as X-Y, where X is the type or subtype/lineage of the first infection and Y is that of the subsequent (consecutive) infection. We defined mixed infections as notifications for different influenza types or subtypes/lineages with onset date within 14 days of the original notification. Identification of mixed A (i.e. co-infection by H1N1 and H3N2) and mixed B (i.e. B/Victoria and B/Yamagata) infections was limited to notifications where subtyping was performed, which is laboratory-dependent. We distinguished between A(H1N1) and A(H1N1)pdm09 notifications as they represented infection by antigenically shifted viruses.

**Fig. 1.**
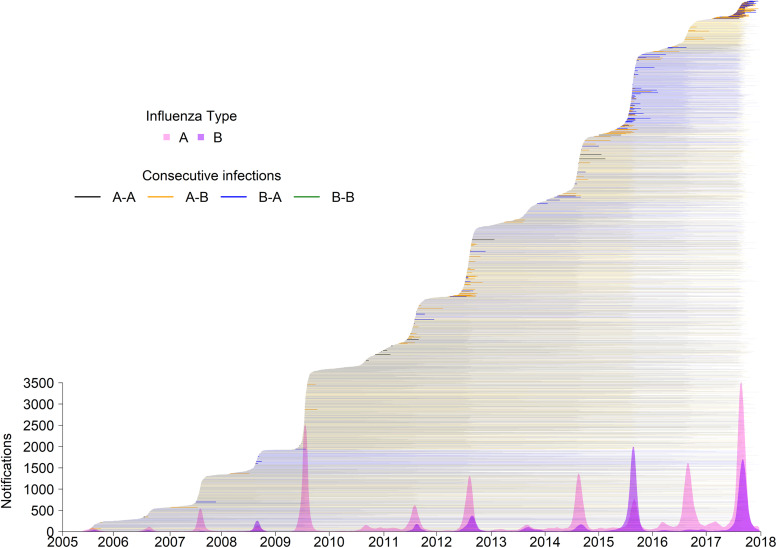
Notifications made in Queensland for the period 2005–2017. *Notes*: Notifications smoothed using 3-week moving average of weekly counts. Line segments indicate the period between reinfection with any influenza type/subtype.

Queensland includes both temperate and tropical climates. However, the majority of the population resides in temperate regions, so the overall temporal trend of notification data follows that of a temperate climate, which in the Southern Hemisphere is observed as a single epidemic peak in the winter months (June–August; Figure 1). Therefore, we have assumed consecutive infections occurring during the same calendar year occurred within the same influenza season.

### Statistical analysis

Epidemic curves were generated using a smoothed three-week moving average of weekly counts. To determine the cumulative incidence of reinfection, we used Kaplan-Meier estimators, which account for right censoring. Cases entered the dataset at the time of their first influenza infection on or after 9 January 2005 and were censored on 31 December 2017. The log-rank test was used to compare the estimators by gender and age group (<5 years, 5–17, 18–64, ≥65). To estimate hazard ratios for reinfection by gender and age group, the Prentice, Williams and Peterson gap time model was used [25]. This modification of the Cox proportional hazards model allows for multiple failures (i.e. repeat influenza infections) by stratifying on an ordered variable that indicates the number of events for which each individual is at risk. For example, an individual in our dataset who was reinfected *k* times enters the dataset at the time of their first influenza infection at risk for one reinfection (event = 1), after their first reinfection they are at risk for a second (event = 2), and so on until the *k*th reinfection, after which they are at risk for the *k* + 1th event. However, estimates using this model become unstable when the risk set becomes too small [26]. Therefore, for the purpose of this analysis, we censored individuals at their third infection (*n* = 27 events excluded). As we used disease notification data, we were unable to stratify analyses by vaccination status. The small number of infections with subtype/lineage information also precluded the use of survival analysis with that granularity; instead, these results are presented descriptively. All data analyses and visualisation were undertaken in R (version 4.0.2), using the *survival* and *survminer* packages [27, 28].

## Results

From 2005 to 2017 there were 188 392 influenza notifications for 182 155 individuals. Individuals contributed 641 562 person-years at risk during the observation period. Notifications per influenza season increased over the study period (Fig. 1). Of total notifications, 73.1% (*n* = 137 660) were type A and 26.9% (*n* = 50 732) were B. Subtype information was available for 23.8% of type A infections, while lineage information was only available for 2.1% of type B infections. The proportion of notifications subtyped was not constant across influenza seasons; it peaked during the 2009 pandemic and remained high the following two years, ranging from 6.2% in 2017 to 66.3% in 2009.

### Consecutive infections over the study period

The distribution of consecutive infections over the study period is shown in Figure 1. Most individuals (96.8%, *n* = 176 236) had only a single notification. Detection of multiple notifications among individuals became more common over time. Among those who experienced more than one influenza infection (*n* = 5919), 95.1% (*n* = 5628) had two infections during the study period, 4.5% (*n* = 269) had three, and <1% (*n* = 22) had four or more. Consecutive infections of type A-A were most common (42.7% of consecutive infections, *n* = 2664), followed by A-B (32.2%, *n* = 2007), B-A (21.9%, *n* = 1369) and B-B (3.2%, *n* = 197).

**Fig. 2.**
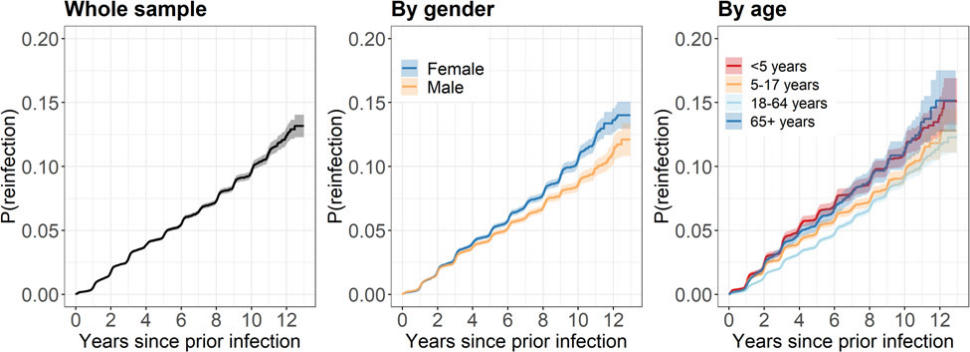
Kaplan-Meier plots displaying cumulative incidence of consecutive influenza infection. *Notes*: P(reinfection): probability (cumulative incidence) of consecutive infection. Shading indicates the 95% confidence interval.

Overall, the cumulative incidence of influenza reinfection one year after a previous infection was <1%, 4.6% after five years, and 9.6% after ten years (Fig. 2). Cumulative incidence differed by gender (*P* = 0.007) and became higher for females than males over time; after one year it was 0.55% and 0.60%, 4.8% and 4.3% after five years, and 10.5% and 8.5% after ten years for females and males, respectively. Accordingly, females had a 1.2-fold risk of consecutive influenza infection compared to males (adjusted hazard ratio (aHR) 1.15, 95% confidence interval (CI) 1.09–1.21).

Cumulative incidence also differed by age (*P* < 0.001) and was highest among those aged <5 years or ≥65 years. Among children aged <5 years at first notification, the cumulative incidence was 0.9%, 6.1% and 11.2% after one, five and ten years, respectively. For 5–17 years it was 0.7%, 4.9% and 10.0%, for 18–64 years it was 0.4%, 3.9% and 9.2%, and for ≥65 years it was 0.7%, 5.6% and 11.4%. Children aged <5 years (aHR 1.58, 95% CI 1.47–1.70), children aged 5–17 years (aHR 1.21, 95% CI 1.14–1.29) and adults ≥65 years (aHR 1.35, 95% CI 1.24–1.47) all had a higher risk of reinfection compared to adults aged 18–64 years.

**Fig. 3.**
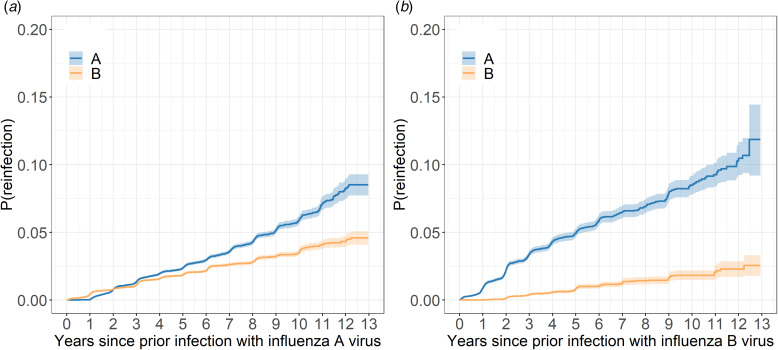
Kaplan-Meier plots displaying cumulative incidence of consecutive influenza infection after infection with (a) influenza type A virus and (b) influenza type B virus. *Notes*: P(reinfection): probability (cumulative incidence) of consecutive infection given prior infection. In these plots, only individuals who were infected with the first influenza type are considered. Shading indicates the 95% confidence interval.

When considering consecutive infections after initial infection with type A, the probability of consecutive infection with type B was higher than that of type A for approximately the first two years after initial infection, whereafter reinfection with type A became more likely (Fig. 3a). After five years the probability of reinfection was 2.4% and 1.9% for type A and B, respectively, and 5.9% and 3.5% after 10 years. After a type B infection, the probability of consecutive infection with type A was always higher than consecutive infection with type B (Fig. 3b).

The distribution of subtypes/lineages causing consecutive infections where subtype/lineage information was available for both infections is described in Table 1. There were 25 instances of consecutive infection by the same subtype (H1N1(pdm09)-H1N1(pdm09): *n* = 9; H3N2-H3N2: *n* = 16). There were no recorded consecutive infections including two B lineages, though B viruses with determined lineage were few. For consecutive infections where both infections were caused by H1N1(pdm09), the median time interval between infections was 735 days (range: 380–2987), while for H3N2 it was 1493 days (400–3416 days).

**Table 1.** Distribution of consecutive notifications from 2005–2017 by subtype/lineage (*N* = 199)

	Second infection				
	H1	H1(pdm09)	H3	B/Victoria	B/Yamagata
First infection					
H1		9	6		
H1(pdm09)		9	92	3	3
H3	1	40	16	1	2
B/Victoria		1	5		
B/Yamagata		7	4		

*Notes*: Only consecutive infections where the subtype/lineage was known for both notifications are shown. **Empty cells indicate no consecutive notifications of those subtypes/lineages.**

### Consecutive infections occurring within the same influenza season

Three-hundred and thirty consecutive infections occurred within the same season (i.e. same calendar year), representing 5.6% of all consecutive infections. The majority were type A-B (63.0%, *n* = 208), followed by B-A (34.5%, *n* = 114), with very few type A-A (2.4%, *n* = 8) and none of type B-B. These consecutive notifications were not prevalent until 2011, and they were more common in years with a higher number of notifications and co-dominance of both type A and B viruses circulating (2012, 2015, 2017). In years when the peak of type B notifications followed the peak of type A notifications, there were more A-B notifications than B-A. However, in the two years that saw A and B peak at similar times (2014 and 2015), there were a similar number of A-B and B-A consecutive infections. Subtype/lineage information was only available for both notifications for three of these consecutive infections.

### Mixed infections

**Fig. 4.**
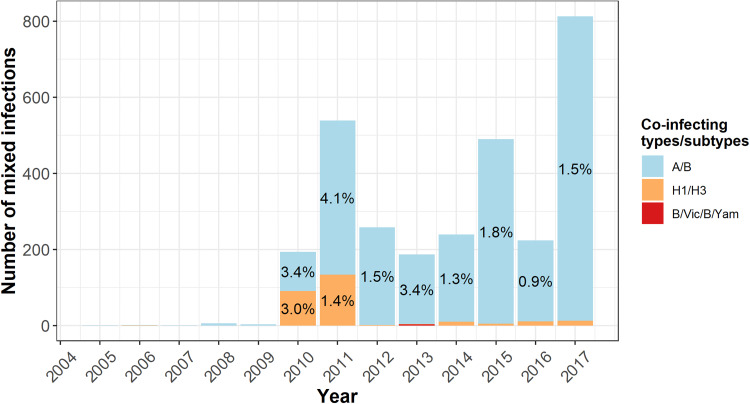
Distribution of mixed influenza infections. *Notes*: Data labels represent the per cent mixed influenza infections represented out of total infections in a given year. To improve visibility of trends, data labels are not shown for percentages <0.9%.

The distribution of mixed influenza infections is shown in Figure 4. Of all mixed infections (*n* = 2958; 1.6% of notifications), the majority were detected in the same specimen (*n* = 2748, 93.0%), with the remaining detected from different specimens with either the same illness onset date or onset dates less than 14 days apart. The majority of mixed infections were type A/B infections (*n* = 2958, 90.8% of total mixed infections). Mixed A/B notifications were uncommon in years prior to 2010, accounting for <0.2% of notifications each year. Mixed infections of the same type (i.e. H1N1-H3N2 or B/Victoria-B/Yamagata) could only be identified when samples were subtyped. There were 270 mixed A infections and three mixed B infections over the study period (Table 1). Only one mixed infection of the same type was observed before 2010. The majority of mixed A infections occurred in 2010 and 2011 (*n* = 225, 85.0%) when the highest proportion of samples was subtyped (data not shown). Mixed H1-H3 infections represented 0.8% of subtyped A notifications. All three mixed B infections occurred in 2015, a year of high influenza B circulation. The median age of people with a mixed infection was 39.0 years (IQR: 17.3–59.9), older than that of all notifications (32.0, IQR: 12.3–54.2).

## Discussion

We observed consecutive infections in 3.3% of notifications. Females and children under five years of age were most likely to experience a consecutive infection. Like previous retrospective analyses of influenza testing data, we found reinfection within the same influenza season of the prior infection to be possible, but rare [6, 29].

Across the entire study period, consecutive infection of type A-A was most common. This is consistent with observations that influenza A viruses undergo antigenic change at a faster rate, exhibit greater diversity [30], and have higher attack rates than the influenza B viruses [31]. However, for the first two years after type A infection, the probability of consecutive infection with type B was more likely than type A. This confirms our expectations that immunity post-influenza infection is more protective against the same type than cross-protective against the other type, limiting opportunities for reinfection. It is also possible sufficient antigenic drift required for reinfection with the same subtype may require multiple influenza seasons to occur. Type B influenza infections are associated with less severe disease in adults [32], which may therefore reduce the propensity to seek healthcare and be counted among notifications. Attenuated severity of the influenza B viruses may explain the lower probability of consecutive infection with type B after type A infection, and also the dearth of B-B infections in our data.

Unlike consecutive infections of any interval, consecutive infections within the same influenza season were more likely to be heterotypic (i.e. A-B or B-A). We found no reinfection with the same subtype or lineage within a year; although the proportion of viruses with subtype or lineage information was small. When the data were expanded to the entire study period, a handful of reinfections with the same subtype were observed, with the period between infections shorter for H3 compared with H1 viruses. This is consistent with our expectations, given the H3 virus undergoes antigenic drift more quickly than H1 and drift variants co-circulate rather than replace existing variants as for H1 [33]. A prospective household study found individuals could be reinfected with A(H3N2) within two-to-five years of initial infection with sufficient antigenic drift of the two infecting viruses [12]. A small study of healthy adults showed that it was possible to infect individuals twice by sequential challenge using the identical A(H1N1)pdm09 strain 7.5–18.5 months apart [34]. However, while 5 of the 7 volunteers showed clinical signs of infection after the second challenge, only 3 had laboratory evidence of reinfection. It is important to note that challenge studies and prospective household studies test for infection in the absence of symptoms; therefore, it is possible that the true number of reinfected individuals is higher than our dataset suggests given it relies on individuals being sufficiently ill to seek healthcare and for their primary care physician to order a test.

Consecutive infections were most common in children aged <5 years, which is unsurprising since these infections may represent the child's first infection with that subtype or lineage. Having been infected once, the propensity for re-infection with the same subtype or lineage may be reduced leading to longer times between infections, consistent with our observations of reduced probability of multiple notifications among adults. Modelling studies have also shown that immunologic protection against H3 infection lasts longer in adults than children after influenza H3 infection [35], which was reflected in our analysis that showed children aged 5–17 years had a higher probability of consecutive infection than adults aged 18–64 years. These observations are consistent with our understanding of increased susceptibility to infection among children, because of immature immunity [36]. Older adults aged ≥65 years were also more likely than adults aged 18–64 years to experience a consecutive influenza infection, which is consistent with our understanding of the effect of immunosenescence on both natural- and vaccine-induced immunity to influenza [37].

Mixed-type infections were observed infrequently over the study period and were particularly uncommon before 2010. This observation is likely to be the result of changes to testing procedures in Australia after the 2009 pandemic, which led to an overall increase in notified infections. This post-pandemic increase was not observed in a Brazilian study of dual and triple influenza infections from 2009 to 2018 [18]. However, as mixed influenza infections have been associated with cardiopathy and death [18], this finding warrants further investigation.

A strength of our study is the long observation period and large number of notifications that could be linked by individual over time. However, the nature of the notification data used in this study led to some limitations. We do not have vaccination information and care should be taken in making inferences about individuals' immune responses to influenza infection without knowing their vaccination status. There is individual variability in the duration of shedding post-influenza infection; therefore, the 14-day cut off we used to denote a new infection may not necessarily be appropriate for all individuals. Several factors we could not account for may have resulted in an underestimation of the cumulative incidence of influenza reinfection. First, reinfection with influenza may result in less severe symptoms [38], which may decrease the chance an individual seeks healthcare and thus, the chance they are tested for influenza. Second, we may have overestimated person-time under observation as we do not know whether individuals remained in Queensland for the entire study period. Third, identification of consecutive infections was based on personal information, which can change between notifications (e.g. name changes) and data entry errors may inhibit reliable identification of sequential observations for individuals. The incomplete subtype/lineage information in our dataset meant we were unable to draw conclusions regarding the prevalence of reinfection by the same subtype. Finally, the use of notification data to infer temporal trends and generalise observations to future seasons is precarious given they lack a denominator and testing patterns have changed over time [39]. This also limits our ability to draw definitive conclusions about protection and cross-protection generated by the individual immune response.

## Conclusion

In this study, we provide epidemiological data to complement immunological studies about the duration of immunological protection post-influenza infection. Our data support others' observations that consecutive influenza infections are possible, but uncommon, especially within the same influenza season. Our findings may inform influenza infection modelling as they have implications for the rate at which recovered individuals become susceptible to disease again. Future studies of consecutive and mixed influenza infections would benefit from more subtype/lineage data.

## Data Availability

Authorisation for the use of human disease data from the Queensland Notifiable Conditions Register was obtained from the Communicable Diseases Branch, Department of Health, Queensland Government.

## References

[1] Paget J (2019) Global mortality associated with seasonal influenza epidemics: new burden estimates and predictors from the GLaMOR Project. Journal of Global Health 9, 020421. 31673337 10.7189/jogh.09.020421 PMC6815659

[2] Krammer F (2018) Influenza. Nature Reviews Disease Primers 4, 3. 10.1038/s41572-018-0002-y PMC7097467 29955068

[3] van de Sandt CE (2015) Influenza B virus-specific CD8+ T-lymphocytes strongly cross-react with viruses of the opposing influenza B lineage. The Journal of General Virology 96, 2061–2073. 25900135 10.1099/vir.0.000156 PMC4681061

[4] Kreijtz JH, Fouchier RA and Rimmelzwaan GF (2011) Immune responses to influenza virus infection. Virus Research 162, 19–30. 21963677 10.1016/j.virusres.2011.09.022

[5] Koutsakos M (2019) Human CD8(+) T cell cross-reactivity across influenza A, B and C viruses. Nature Immunology 20, 613–625. 30778243 10.1038/s41590-019-0320-6

[6] Arnott A (2017) Consecutive influenza infections in both adults and children. The Journal of Infectious Diseases 215, 658–659. 28329041 10.1093/infdis/jix016

[7] Most J, Redlberger-Fritz M and Weiss G (2019) Multiple influenza virus infections in 4 consecutive epidemiological seasons: a retrospective study in children and adolescents. Open Forum Infectious Diseases 6, ofz195. 31223630 10.1093/ofid/ofz195 PMC6579483

[8] Frank AL (1979) Reinfection with influenza A (H3N2) virus in young children and their families. The Journal of Infectious Diseases 140, 829–836. 541521 10.1093/infdis/140.6.829

[9] Frank AL, Taber LH and Wells JM (1983) Individuals infected with two subtypes of influenza A virus in the same season. The Journal of Infectious Diseases 147, 120–124. 6822747 10.1093/infdis/147.1.120

[10] Sonoguchi T (1986) Reinfection with influenza A (H2N2, H3N2, and H1N1) viruses in soldiers and students in Japan. The Journal of Infectious Diseases 153, 33–40. 3941288 10.1093/infdis/153.1.33

[11] Nakajima S (1995) Analysis of influenza A virus reinfection in children in Japan during 1983–91. Epidemiology and Infection 115, 591–601. 8557091 10.1017/s0950268800058751 PMC2271582

[12] Smith CB (2002) Molecular epidemiology of influenza A(H3N2) virus reinfections. Journal of Infectious Diseases 185, 980–985. 11920323 10.1086/339416

[13] Horby P (2012) The epidemiology of interpandemic and pandemic influenza in Vietnam, 2007–2010: the Ha Nam household cohort study I. American Journal of Epidemiology 175, 1062–1074. 22411862 10.1093/aje/kws121 PMC3353138

[14] Perez CM, Ferres M and Labarca JA (2010) Pandemic (H1N1) 2009 reinfection, Chile. Emerging Infectious Diseases 16, 156–157. 20031070 10.3201/eid1601.091420 PMC2874388

[15] Trakulsrichai S, Watcharananan SP and Chantratita W (2012) Influenza A (H1N1) 2009 reinfection in Thailand. Journal of Infection and Public Health 5, 211–214. 22541272 10.1016/j.jiph.2011.10.010

[16] Freeman G (2016) Quantifying homologous and heterologous antibody titre rises after influenza virus infection. Epidemiology and Infection 144, 2306–2316. 27018720 10.1017/S0950268816000583 PMC5530596

[17] Szymanski K (2017) Co-infection with influenza viruses and influenza-like virus during the 2015/2016 epidemic season. Advances in Experimental Medicine and Biology 968, 7–12. 28181195 10.1007/5584_2016_182 PMC7122344

[18] Gregianini TS (2019) Dual and triple infections with influenza A and B viruses: a case-control study in southern Brazil. The Journal of Infectious Diseases 220, 961–968. 31125400 10.1093/infdis/jiz221

[19] Topoulos S (2019) Analysis of acute respiratory infections due to influenza virus A, B and RSV during an influenza epidemic 2018. Infection 47, 425–433. 30649684 10.1007/s15010-018-1262-x

[20] Calistri A (2011) Report of two cases of influenza virus A/H1N1v and B co-infection during the 2010/2011 epidemics in the Italian Veneto Region. Virology Journal 8, 502. 22050693 10.1186/1743-422X-8-502 PMC3233533

[21] Falchi A (2008) Dual infections by influenza A/H3N2 and B viruses and by influenza A/H3N2 and A/H1N1 viruses during winter 2007, Corsica Island, France. Journal of Clinical Virology: The Official Publication of the Pan American Society for Clinical Virology 41, 148–151. 18069055 10.1016/j.jcv.2007.11.003

[22] Pando R (2017) Influenza A(H1N1)pdm 2009 and influenza B virus co-infection in hospitalized and non-hospitalized patients during the 2015–2016 epidemic season in Israel. Journal of Clinical Virology: The Official Publication of the Pan American Society for Clinical Virology 88, 12–16. 28088665 10.1016/j.jcv.2017.01.002

[23] Fielding JE (2014) Systematic review of influenza A(H1N1)pdm09 virus shedding: duration is affected by severity, but not age. Influenza and Other Respiratory Viruses 8, 142–150. 24299099 10.1111/irv.12216 PMC4186461

[24] Carrat F (2008) Time lines of infection and disease in human influenza: a review of volunteer challenge studies. American Journal of Epidemiology 167, 775–785. 18230677 10.1093/aje/kwm375

[25] Prentice RL, Williams BJ and Peterson AV (1981) On the regression analysis of multivariate failure time data. Biometrika 68, 373–379.

[26] Amorim LD and Cai J (2015) Modelling recurrent events: a tutorial for analysis in epidemiology. International Journal of Epidemiology 44, 324–333. 25501468 10.1093/ije/dyu222 PMC4339761

[27] Therneau TM and Lumley T (2014) Package ‘survival’. Survival Analysis 2, 119.

[28] Kassambara A (2017) Survminer: drawing survival curves using ‘ggplot2’. In: The Comprehensive R Archive Network. https://cran.microsoft.com/snapshot/2017-04-21/web/packages/survminer/survminer.pdf.

[29] Most J and Weiss G (2016) Consecutive infections with influenza A and B virus in children during the 2014–2015 seasonal influenza epidemic. The Journal of Infectious Diseases 214, 1139–1141. 27095422 10.1093/infdis/jiw104

[30] Bedford T (2014) Integrating influenza antigenic dynamics with molecular evolution. Elife 3, e01914. 24497547 10.7554/eLife.01914 PMC3909918

[31] Jayasundara K (2014) Natural attack rate of influenza in unvaccinated children and adults: a meta-regression analysis. BMC Infectious Diseases 14, 670. 25495228 10.1186/s12879-014-0670-5 PMC4272519

[32] Koutsakos M (2016) Knowns and unknowns of influenza B viruses. Future Microbiology 11, 119–135. 26684590 10.2217/fmb.15.120

[33] Carrat F and Flahault A (2007) Influenza vaccine: the challenge of antigenic drift. Vaccine 25, 6852–6862. 17719149 10.1016/j.vaccine.2007.07.027

[34] Memoli MJ (2019) Influenza A reinfection in sequential human challenge: implications for protective immunity and ‘universal’ vaccine development. Clinical Infectious Diseases: An Official Publication of the Infectious Diseases Society of America 70, 748–753. 10.1093/cid/ciz281 PMC7319262 30953061

[35] Ranjeva S (2019) Age-specific differences in the dynamics of protective immunity to influenza. Nature Communications 10, 1660. 10.1038/s41467-019-09652-6 PMC6458119 30971703

[36] Sullivan SG, Price OH and Regan AK (2019) Burden, effectiveness and safety of influenza vaccines in elderly, paediatric and pregnant populations. Therapeutic Advances in Vaccines and Immunotherapy 7. https://journals.sagepub.com/doi/full/10.1177/2515135519826481. 10.1177/2515135519826481 PMC6376509 30793097

[37] McElhaney JE (2020) The immune response to influenza in older humans: beyond immune senescence. Immunity & Ageing: I & A 17, 10. 32399058 10.1186/s12979-020-00181-1 PMC7204009

[38] Davies JR, Grilli EA and Smith AJ (1984) Influenza A: infection and reinfection. Journal of Hygiene (London) 92, 125–127. 10.1017/s002217240006410x PMC2129348 6693762

[39] Fielding JE (2016) How severe was the 2015 influenza season in Australia? The Medical Journal of Australia 204, 60–61. 26821099 10.5694/mja15.01094

